# The macrophages regulate intestinal motility dysfunction through the PGE2 Ptger3 axis during Klebsiella pneumonia sepsis

**DOI:** 10.3389/fimmu.2023.1147674

**Published:** 2023-03-29

**Authors:** Hua Yao, Xin Fu, Qian Xu, Tingting Li, Yao Li, Yan Kang, Qin Wu

**Affiliations:** Department of Critical Care Medicine, West China Hospital, Sichuan University, Chengdu, China

**Keywords:** macrophages regulate intestinal motility dysfunction sepsis, immunity, neutrophils, muscular neurons, intestinal muscular macrophages

## Abstract

**Introduction:**

Gut motility dysfunction, the most common complication of post-septic organ dysfunction, depends on immune and neuronal cells. This study aimed to investigate the mechanisms that activate these cells and the contribution of macrophages to the recovery of intestinal motility dysfunction after sepsis.

**Materials and methods:**

Postoperative gut motility dysfunction was induced by establishing Klebsiella pneumonia sepsis in mice with selective deletion of neutrophils and macrophages in the gut. The distribution of orally administered fluorescein isothiocyanate-dextran and carmine excretion time was used to determine the severity of small bowel disease. The effect of macrophages on intestinal motility was evaluated after prostaglandin E2 therapy.

**Results:**

We found that muscular neutrophil infiltration leading to neuronal loss in the intestine muscle triggered intestinal motility dysfunction after pneumonia sepsis; however, reduced neutrophil infiltration did not improve intestinal motility dysfunction. Moreover, macrophage depletion aggravated gut motility dysfunction. The addition of macrophages directly to a smooth muscle was responsible for the recovery of intestinal motility.

**Conclusion:**

Our results suggest that a direct interaction between macrophages and smooth muscle is neurologically independent of the restoration of intestinal dysmotility.

## Graphical Abstract

Illustration of normal intestine motility and sepsis intestine motility. CSF1: Colony stimulating factor 1, GPCRs: G-protein-coupled receptors. Ptger3: Prostaglandin E Receptor 3. Created with BioRender.com.
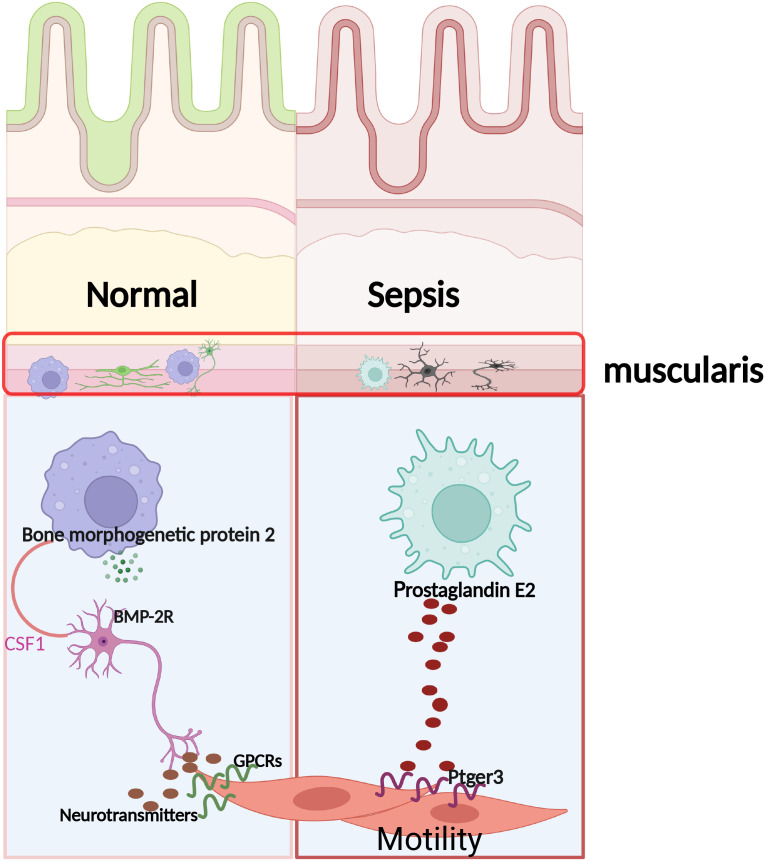

## 1 Introduction

Klebsiella pneumonia (KP) is a common leading cause of sepsis in humans and is characterized by high morbidity and mortality (1). Notably, Klebsiella bacteria causes pneumonia, urinary tract infections, and bloodstream infections, among other diseases (2). The mouse model of pneumonia replicating manifestations of KP in humans is being used to explore related diseasemechanisms, including massive inflammation, edema, and an influx of polymorphonuclear neutrophils (3).

Intestinal dysfunction contributes significantly to the development of potentially fatal infections and multiorgan dysfunction (4). Therefore, the development of intestinal dysmotility in sepsis is recognized as a major complication. Although several intestinal dysfunction pathogenic mechanisms caused by sepsis have been proposed, the mechanisms underlying this process are poorly understood.

Smooth muscle contraction or relaxation is widely known to play a crucial role in gastrointestinal motility (5). However, the immune and nervous systems of the gut have been reported to detect and integrate intraluminal signals to regulate physiological processes, including gastrointestinal motility.

Furthermore, neutrophils are the most abundant subset of leukocytes in the bloodstream. They are the first line of defense of the host during tissue injury or infection (6). The induction of a pro-inflammatory environment within the injured tissue results in an influx of neutrophils and monocytes (7). For example, in mice with experimental colitis. Neutrophils impair intestinal permeability, causing bacterial translocation to generate inflammation, In addition, they induce epithelial cell apoptosis and disrupt the integrity of tight junctions and adherent junctions, resulting in colon motility dysfunction (8). However, macrophages have many properties and play vital roles in tissue homeostasis throughout the body (9). It has been reported that muscularis macrophages (MΜϕs) are a particular subpopulation of intestinal macrophages, anatomically and physically associated with the myenteric plexus (10). In postoperative ileus, IL-10 produced by intestinal MΜϕs affects the migration of neutrophils to the intestinal muscularis by regulating the expression of neutrophil chemokines, contributing to the healing process of postoperative ileus and solving the occurrence (11).

The transient receptor potential (TRP) family in the gastrointestinal tract is highly expressed and can regulate physiological functions, such as gastrointestinal motility, visceral secretion, and visceral hypersensitivity (12). TRP channels are a superfamily of transmembrane cation channels; TRPC (Canonical), TRPV (Vanilloid), TRPA (Ankyrin), TRPM (Melastatin), TRPML (Mucolipin), and TRPP (Polycystin) are the six subfamilies of this superfamily, the majority of which are related to Ca2+ influx (13). They are triggered by endogenous, chemical, mechanical, thermal, osmotic, and other signals and may be crucial regulators of various gastrointestinal tract functions (14). Notably, TRPV1 is reportedly activated by submucosal plexus-expressed capsaicin and associated with stress-induced visceral hypersensitivity (15). Inspired by the co-stimulatory properties of these natural products, investigating whether sepsis stimulates gastrointestinal motility *via* a similar mechanism would be worthwhile.

## 2 Materials and methods

### 2.1 Animals

The Dashuo Laboratory (Chengdu, China) provided C57BL/6 specific pathogen-free WT female mice bred at the West China Hospital Frontiers Science Center for Disease-related Molecular Network following HuaXi guidelines. In addition, all animal experiments were approved by the Institutional Animal Care and Use Committee, West China Hospital (study number:20220211010). Neutrophils were exhausted using the anti- Ly6g mAb RB6-8C5 (BioXCell, New Hampshire, USA). Furthermore, mice were intravenously administered mouse anti-Ly6gmAb RB6-8C5 (0.1 mg/day) a day before KP infection to deplete neutrophils. Meanwhile, 200 µL of clodronate-containing liposomes were injected intravenously to deplete MΜϕs (YEASEN, Shanghai, China) 24 h before KP infection. Finally, flow cytometry was used to confirm the effectiveness of neutrophil and macrophage depletion in the KP-infected mice.

### 2.2 Pneumonia lung infection

KP strain was cultivated in tryptic soy broth at 37°C for 16 h. Subsequently, 750 μL of the tryptic soy broth culture was added to 30 mL of fresh broth, and the organism was grown for an additional 2 h to achieve log phage. The bacteria were pelleted for 10 min by centrifugation at 4,500 rpm, washed in cold sterile normal saline, and resuspended at the desired concentration. Subsequently, the absorbance at 600 nm was used to calculate KP concentration. Finally, the mice were briefly sedated with isoflurane pre-inoculation. The bacterial inoculum (30 μL) was applied using a pipette tip to the trachea of a mouse and was involuntarily inhaled.

### 2.3 RNA isolation and blood enzyme-linked immunosorbent assay

Total RNA samples were isolated from intestine muscularis using TRIzol reagent (Invitrogen, Thermo Fisher Scientific). Briefly, separating intestine muscularis and using liquid nitrogen quick freezing, then extracted with ethanol, isopropanol, and chloroform. A Thermo Fisher Scientific Superscript III Reverse Transcriptase kit was used to create cDNA, and RT-PCR was performed using a Bio-Rad machine. The data were subjected to relative quantification using 18S ribosomal RNA (2–ΔΔCt) as a control. Each sample was analyzed thrice. A list of RT-qPCR primers used is provided in **Table 1.** At 12 hours and 1 day after the control or KP sepsis operation (n = 6 per group), mice were euthanasia and rapidly harvested blood samples. Blood samples clot for 30 min at room temperature and then centrifugation for 15 min at 1000 g. Subsequently the supernatants were saved at −80 °C for detection. Blood supernatant protein concentration was measured by enzyme-linked immunosorbent assay kits for IL-1β (R0201-1, Nuohe Bio, Chengdu, China), IL-6 (R0201-6, Nuohe Bio, Chengdu, China), lactic acid (R0201-19, Nuohe Bio, Chengdu, China), TNFα (R0201-8, Nuohe Bio, Chengdu, China), and CCL5 (R0201-12, Nuohe Bio, Chengdu, China) to detect expression levels. These assays were carried out according to the instructions provided by the manufacturer. Each sample was detected twice at least.

**Table 1:** Primer sequences of mouse Ccl5 and Actg1.

	CCL5	Ptger3
Forward Primer	GCTGCTTTGCCTACCTCTCC	CCGGAGCACTCTGCTGAAG
Reverse Primer	TCGAGTGACAAACACGACTGC	CCCCACTAAGTCGGTGAGC

### 2.4 Measurement of small intestinal transit

Each mouse received an oral dose of carmine (50 μL; 3 mg of carmine in 0.5% methylcellulose). Subsequently, the mice were put back in separate cages and placed on a blank piece of paper, the duration for the first red feces to be excreted was noted. Furthermore, five to ten animals of each genotype were used to measure the gut transit time.

### 2.5 Flow-cytometry

The muscularis layers of 9 or 12 mice per group were harvested for further analyses to collect enough intestinal muscularis propria neutrophils and macrophage cells. A single-cell suspension was prepared as described previously (16). Briefly, the intestinal muscularis layer tissue was cut into 1-2 mm, and added to 8 mL HBSS (Gibco, America) of digestion solution containing 10 mg/L II collagenase (9001-12-1, Sigma, Germany), 2.4mg/mL II dispase (D6430, Solarbio, Beijing, China), 0.1mg/mL DNase I (D8071, Solarbio, Beijing, China), 1 mg/mL bovine serum albumin(V900933, Sigma, Germany), and 0.7 mg/L soybean trypsin inhibitor (T8031, Solarbio, Beijing, China). Furthermore, the resulting solution was put in a constant temperature water bath at 37°C for 15 min to see the intestinal segment become transparent, and add 10 mL of HBSS (Gibco, America) containing 5% BSA was added to stop digestion. Next, it was passed through a 70-mesh sieve and centrifuged at 400 g/4°C for 7 min, then use 10 mL of DPBS washing buffer containing 0.04% BSA was used twice. Approximately, 10 mL of DMEM (Gibco, America) culture solution was used to resuspend the cells. Finally, the cells were stained for 30 min at 4°C with the relevant antibodies after incubation with FcR-block (anti-CD16/32, BioLegend). The antibodies used in this study are listed in **Table 2**. Different cell populations were captured in PBS with 10% FCS after cell sorting was completed on the FACSAria III (BD Bioscience) platform.

**Table 2:** Sources of commercial antibodies used in flow-cytometry experiments.

Specificity	Fluorochrome	Cat#	Supplier
CD45	APC	103112	BioLegend
CD11b	PE-Cy7	101216	BioLegend
LY6G	PerCP/Cy5.5	127672	BioLegend
LY6C	PE	128008	BioLegend
MHCII	FITC	107606	BioLegend
DAPI	BV421	C1002	Beyotime

### 2.6 Immunofluorescence staining and transmission electron microscopic

We gently separated the smooth muscle from the mucosal layer using microscopic forceps. One part of muscularis propria was fixed in 0.1 M phosphate buffered saline containing 4% paraformaldehyde for 4 h at 4°C. After washing in PBS containing 5% BSA thrice, the muscular tissue of each group was blocked in blocking solution (PBS containing 0.3% Triton X-100 (Beyotime), 5% normal donkey serum (Beyotime), and 5% BSA (Sigma)) for 1 h at room temperature and then incubated at 4°C for 72 h with primary antibody. **Table 3** shows the primary antibodies and secondary antibody staining. Secondary antibody staining was performed for 2 h. Subsequently, after washing three times with PBS containing 5% BSA, the nuclei were labeled with Beyotime’s DAPI for 5 min. For each sample, at least five fields were blindly evaluated. Another part of muscularis propria was fixed at 4°C in 0.1 M phosphate buffered 3% glutaraldehyde and postfixed in 1% osmium tetroxide in the same buffer. After fully rinsed in distilled water, the samples were dehydrated in graded acetone series and embedded in SPI-Pon812. Ultrathin sections were cut with a Leica EM UC7 ultramicrotome, and attached to copper grids with Formvar film, then stained with 2% uranyl acetate and Reynolds lead citrate and examined under a Hitachi HT7800 electron microscope at 80 kV.

**Table 3:** Sources of commercial antibodies used in immunofluorescence experiments.

Specificity	Cat#	Supplier
HUC/D	A-21271	thermofisher
GFAP	A14673	Abclonal
CD117/ckit	A0357	Abclonal
Ptger3	ab21227	abcam
Caspase3	ab104787	abcam
DAPI	C1002	Beyotime
Tunnel	C1086	Beyotime
Goat Anti-Rabbit IgG H&L	ab150077	abcam
Goat Anti-Mouse IgG H&L	ab150118	abcam
Goat Anti-Rabbit IgG H&L	Ab150083	abcam

### 2.7 Small intestine organ bath experiment

Mice were sacrificed *via* cervical dislocation, and the small intestine was excised completely and immediately transferred to a cold Krebs solution (Solarbio, China). The contents of the intestinal lumen were removed by gently flushing the lumen with Krebs solution using a syringe. Subsequently, the same small intestinal site (2–3 cm) from different groups were carefully mounted longitudinally and gently suspended using cotton thread from force transducers before being submerged in 10 mL organ bath chambers filled with warmed 37°C Krebs solution and gassed with 95% O_2_ and 5% CO_2_. A dedicated data collection system was used to record changes in the tension of the sensor after amplification and processing.

### 2.8 Isolation of primary intestinal SMCs and measurement of the cellular contractile response

The small intestinal muscularis layer of the sepsis mouse model was peeled off and placed in an ice-cold modified Krebs solution (G0430, Solarbio, Beijing, China). The tissue was cut into 1-2 mm small pieces and digested in 6 mL Ca2+ free DPBS solution containing 100 mg/mL collagenase II (001-12-1, Sigma, Germany), 100 mg/mL collagenase IV (C8160, Solarbio, Beijing, China), 1 mg/mL soybean trypsin inhibitor (T8031, Solarbio, Beijing, China), and 1 mg/mL bovine serum albumin (V900933, Sigma, Germany). The resulting solution was shaken at 37°C for 30 min. After digestion, DPBS buffer containing 5% fetal bovine serum was double diluted to stop the digestion and gently blown repeatedly with a dropper. The solution was then centrifuged at 1 000 r/min for 3 min. Next, 10 mL of DMEM/F12 culture solution containing 10% fetal bovine serum was used to resuspend the cells. Furthermore, the sample was passed through a 100-mesh sieve, and centrifuged at 1000 rpm/4°C for 7 min. The cell filtrate was taken and stained with trypan blue to check the cell viability and confirm that the viable cells were above 90%. Moreover, after 24 h of DMEM/F12 culture, the medium was changed, and the non-adherent cells were discared. DMEM/F12 culture medium was added to continue culturing until used for experiments. Each group of cells (Sep7d, Sep7d + PGE2, Sep 7d +PGE2 +EP3 antagonist) was loaded with the Fluo-4 (IF1500, Solarbio, Beijing, China) for 30 min in an incubator under 37°C and 5% CO2 and subsequently washed gently using iced DPBS. Finally, using an experimental device (Leica Stellaris Microsystems, Germany) that combines immunofluorescence and live cell imaging to capture images, the contraction response of smooth muscle cells was expressed by the change of intracellular fluorescence intensity (FI) after using PGE2 and EP3 antagonist to represent the relative concentration change of intracellular free calcium, and analyzed by ImageJ (NIH, Bethesda).

### 2.9 Statistical analyses

All statistical analyses were performed using GraphPad Prism version 8.0(GraphPad Software, Inc., San Diego, CA). We compared the results of the sepsis and control groups using a T-test and one-ANOVA to assess the differences between multiple groups. Differences between groups were significant at a P value of <0.05. * Represents p <0.001, ** represents p <0.01, and *** represents p <0.001. All results are shown as mean ± standard deviation.

## 3 Results

### 3.1 Alterations of intestinal motor dysfunction of KP-infected mice

**Figure 1:**
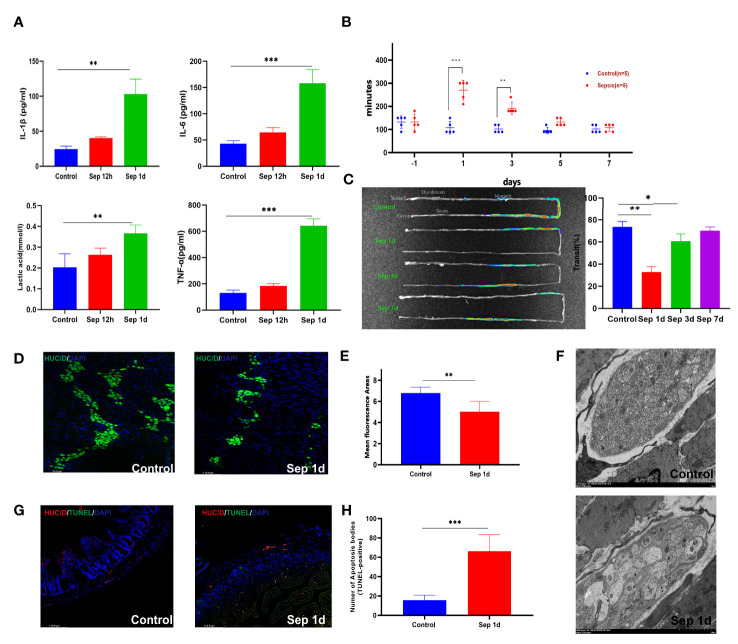
Changes after manipulation of Klebsiella pneumonia (KP)-sepsis mice. **(A)** Changes in plasma IL-6, IL-1β, TNF-α, and lactate levels at 12 and 24 h after KP sepsis. **(B)** The carmine red experiment detected changes in intestinal transit time between the control and KP sepsis groups. **(C)** *In vivo* fluorescence imaging of fluorescein 5-isothiocyanate (FITC)-dextran visually displays intestinal motility changes and histograms of statistical results. **(D, E)** Control and sepsis 1 day after intestinal whole-mount HUC/D fluorescent staining and statistical histogram of neuron fluorescence area changes. **(F)** Transmission electron micrographs of mouse intestinal muscular layer neurons 1 day after control and KP sepsis. **(G, H)** Control and sepsis 1 day of intestine transverse HUC/D and terminal deoxynucleotidyl transferase dUTP nick end labeling (TUNEL) double fluorescent staining and statistical histogram of neuronal apoptosis. Differences between groups were significant at a P value of <0.05. * Represents p <0.05, ** represents p <0.01, and *** represents p<0.001.

The development of KP sepsis-induced intestinal dysmotility dysfunction involves multiple pathogenic mechanisms. Therefore, expanding and deepening our understanding of host defenses and developing new therapeutic strategies is imperative. First, compared with uninfected mice, IL-6, IL-1β, TNF-α, and lactic acid levels in KP-infected mice increased significantly starting at 12 h (**Figure 1A**), and the lung wet-to-dry weight ratio after 1 day. In addition, the pathological sections changed significantly, and the infiltration of neutrophils and monocytes increased (**Figures S1A–C**). Moreover, the clinical score in the sepsis group increased; however, the body weight decreased significantly, with approximately 10% weight loss compared with that of the control group (**Figures S1D–F**).

Gastrointestinal transit was significantly delayed in sepsis mice compared with that in the control group. The total small intestine transit time in the control group was similar to that in the sepsis group pre-operation; however, carmine red and fluorescence *in vivo* imaging showed that the total transit time increased after sepsis (**Figures 1B, C**). The gastrointestinal wall musculature comprises an inner circular and an outer longitudinal smooth muscle layer, both essential for the gastrointestinal tract’s contractile functionality (17). Our result showed that the thickening of the muscular layer of the intestine differs between the control and sepsis groups (con vs. sep1d: 17.42 ± 2.348 vs. 65.28 ± 21.45 μm, *P <*0.001; con vs. sep3d: 17.42 ± 2.348 vs. 101.5 ± 13.89 μm, *P <*0.001; con vs. sep7d: 17.42 ± 2.348 vs. 88.28 ± 17.31 μm, *P <*0.001) (**Figures S1G, H**). In addition to smooth muscle contractile activity, the generation of intestinal contractile forces is further controlled by the enteric nervous system, which comprises of interstitial cells of Cajal (ICCs) and the myenteric plexus (18). We stained adult murine myenteric neurons with a HUC/D antibody, which has been previously shown to mark intestinal neurons (19). We observed a significant loss of HUC/D+ neurons areas by day 1 (mean ± SE of percent HUC/D+ neurons areas 5.52 ± 1.58 vs. 6.73 ± 1.36 at sepsis 1 day vs. control 1 day, from 5 adult mice) (**Figures 1D , E**). c-Kit immunofluorescence staining of intestinal cells of ICCs and Glial fibrillary acidic protein immunofluorescence staining of intestinal glial cells showed no significant differences between the two groups (**Figures S1I, J**). Transmission electron microscopic images of septic enteric neurons showed swelling, rupture, and vacuolation (**Figure 1F**). To determine the impact of lung infections on enteric neuron loss, we quantified enteric neuron loss with HUC/D and tunnel double staining according to the manufacturer’s protocol (Beyotime). Consequently, we found that the number of apoptotic bodies (TUNEL-positive) increased in the sepsis group (con vs. sep1d: 15.64 ± 5.203 vs. 66.25 ± 17.01, *P* =0.002) (**Figures 1G, H**).

### 3.2 Upregulated expression of neutrophils contributes to myenteric neurons loss

**Figure 2:**
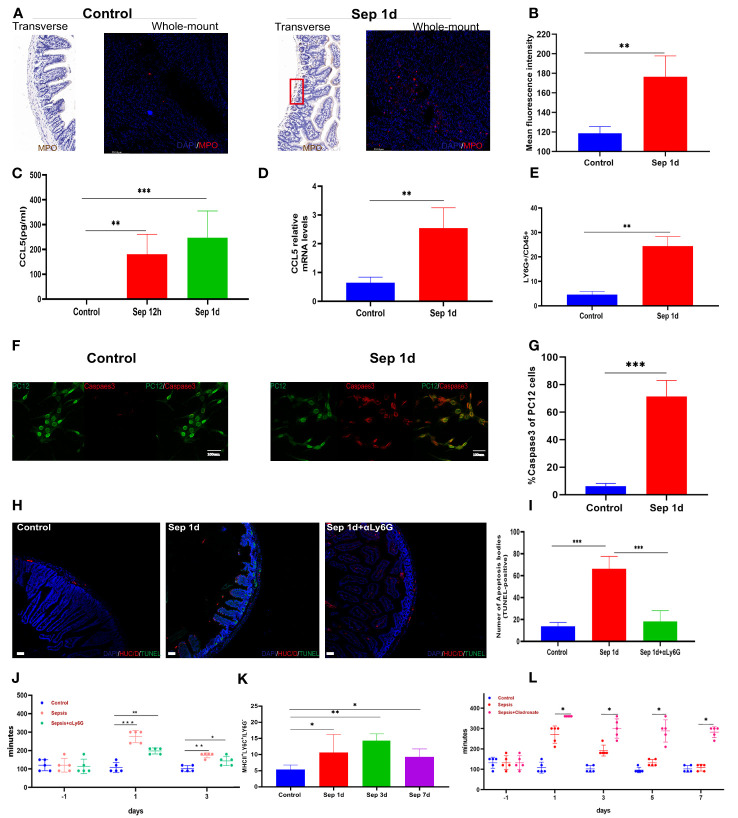
Up-regulates the expression of neutrophils. **(A, B)** Control and sepsis on day 1 of intestine transverse and intestine whole mount myeloperoxidase immunohistochemistry, immunofluorescence staining, and statistical histogram of myeloperoxidase fluorescence intensity changes. **(C)** Changes of plasma CCL5 levels 12 and 24 h after KP sepsis. **(D)** Changes in CCL5 mRNA levels in the intestinal muscularis **(E)**: LY6G^+^ neutrophils in the intestinal muscularis 24 h after KP sepsis. **(F, G)** Immunofluorescence staining of PC12-GFP transgenic cells after co-culture with bone marrow neutrophils and percentages of Caspase3-positive cells. **(H, I)** Control, sepsis 1 day, and neutrophil depletion sepsis 1 day of intestine transverse HUC/D and terminal deoxynucleotidyl transferase dUTP nick end labeling (TUNEL) double fluorescent staining and statistical histogram of neuronal apoptosis. **(J)** The carmine red experiment detects changes in intestinal transit time between the control, KP sepsis, and neutrophil depletion sepsis groups. **(K)** The histogram of statistical changes in MHCII^hi^ expressing cell populations shows the changes in MΜϕs detected by flow cytometry experiments. **(L)** The carmine red experiment detects changes in intestinal transit time between the control group and KP sepsis and macrophage depletion sepsis groups. Differences between groups were significant at a P value of <0.05. * Represents p <0.05, ** represents p <0.01, and *** represents p<0.001.

Neutrophil activation is required for tissue infiltration, contributing to inflammatory responses (20), whereas exaggerated activation and uncontrolled infiltration of neutrophils may cause sepsis (21). Meanwhile, *Mashkaryan* and colleagues identified that macrophages in the brain cause a chronic inflammatory environment and exacerbate neuronal loss in Alzheimer’s disease (22). Moreover, an enteric neuron programmed death has been demonstrated to be closely related to gastrointestinal motility disorder (23, 24). The early onset of neuronal loss led us to examine immune cells in the inflamed small intestine. By immunohistochemistry and immunofluorescence methods, we found that myeloperoxidase expression significantly increased in sepsis on day 1 (**Figures 2A, B**), higher levels of CCL5 occurred at the plasma level as early as 12 h post-sepsis, and CCL5 mRNA levels increased in parallel with those in the muscularis (**Figures 2C, D**). Since we detected a systemic increase in the amounts of myeloperoxidase and CCL5, which are known to be associated with neutrophils, we further detected changes in neutrophils in the intestinal muscularis using Ly6G-positive cells by flow cytometry; this showed a significant increase in the proportion of neutrophils at the time of the most severe intestinal motility dysfunction, which was the first day after KP infection (**Figure 2E**). We further assessed whether neutrophils induce apoptosis in neurons, using flow cytometry to sort out bone marrow neutrophils and resuspend them to a concentration of 10^6^ neutrophils/mL. After 24 h of co-culture with the neuronal cell line PC12-GFP transgenic cells, cells were stained for immunofluorescence, and the percentages of caspase3-positive cells were determined. The results showed a marked increase in the caspase3 percent in sepsis 1-day (**Figures 2F, G**). These results show that enteric neuron loss is caused by the recruitment and activation of pro-inflammatory neutrophils. Subsequently, we sought to determine whether neutrophil blockade could help restore gut motility earlier. **Figures 2H–J** showed that neutrophil depletion *in vivo* reduces apoptosis of intestinal muscularis neurons; however, it had no significant effect on sepsis-induced intestinal motility and was insufficient to restore intestinal motility. In contrast, we observed that a population of MHCII^hi^ -expressing cells emerged from sepsis after 3 days (**Figure 2I**). MΜϕs is a newly discovered population of immune cells exclusively populated by MHCII^hi^ CX3CR1^hi^ that express low levels of CD11c (25). MΜϕs have a different transcriptome compared with mucosal Μϕs, underlying a unique function. Muller et al. discovered that MΜϕs modulate gastrointestinal motility through direct intercommunication with enteric neurons (26). Clodronate-containing liposomes-mediated depletion of macrophages significantly increased animal mortality and aggravated dysmotility (**Figure 2J**). These findings indicated that in KP sepsis, macrophages might be essential for efficient recovery of intestinal motility, with neutrophils playing an insignificant role.

### 3.3 Muscle contraction caused by macrophages specific TRPV4 expression stimulation requires PGE2

In addition to MΜϕs interaction with neurons, one recent study has shown that MΜϕs can directly interact with SMCs by releasing prostaglandin E2 (PGE2), affecting colonic motility(27). Previous studies have demonstrated that nitric oxide and PGE2, two crucial inflammatory mediators released by activated intestinal macrophages, regulate GI motility (28).

**Figure 3:**
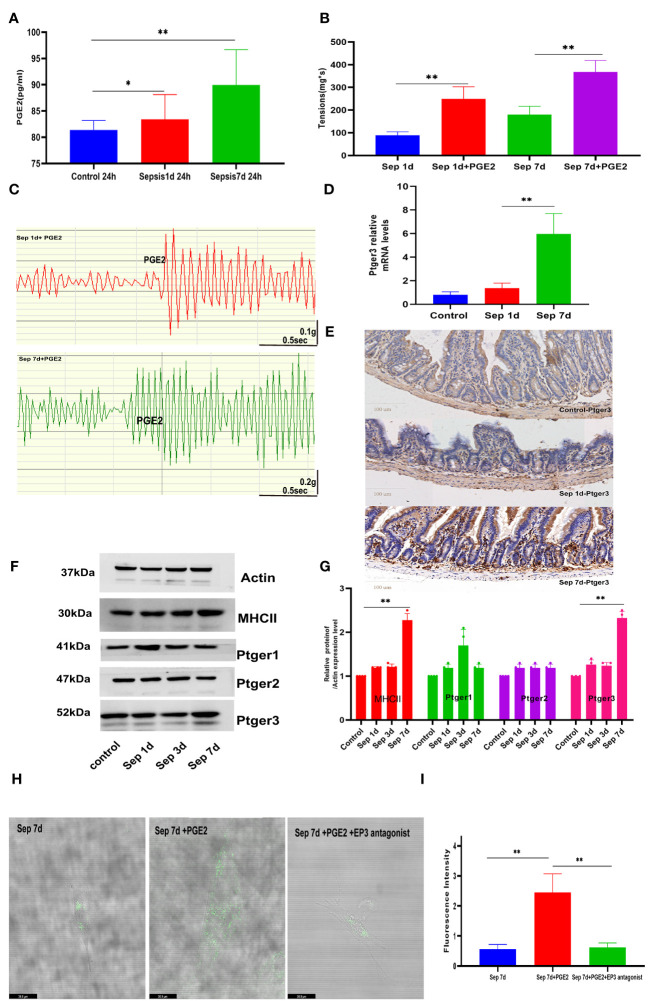
Macrophage-mediated small intestine contraction is prostaglandin E2 (PGE2)-dependent **(A)** Compared sepsis 7 days in mice to sepsis 1 day, PGE2 release from MΜϕs increased in response to GSK101(TRPV4 agonist) stimulation. **(B, C)** Exogenous PGE2 increased the spontaneous contraction force and histogram of the statistical. **(D, E)** Changes in intestinal muscularis Ptger3 mRNA levels and intestinal transverse Ptger3 immunohistochemical staining after control, KP sepsis 1 day and KP 7 days. **(F, G)** E-prostaglandin receptor (EP1–EP3) protein expression was measured by western blotting and histogram of statistical analysis. **(H, I)** The effects of PGE2 and ptger3 antagonists on primary smooth muscle were determined by fluorescence analysis using the fluorescent probe Fluo-4 and histogram of fluorescence intensity statistical analysis. Differences between groups were significant at a P value of <0.05. * Represents p <0.05, ** represents p <0.01, and *** represents p<0.001.

We determined whether macrophage- specific TRPV4 and PGE2 play a similar role in promoting GI movement in pneumonia sepsis. To determine whether TRPV4 signaling on MΜϕs could cause macrophage cells to produce PGE2 directly, we used flow cytometry to separate MHCII^hi^ cells from the muscularis externa, activated them for 24 h with GSK101 (TRPV4 agonist), and then collected the cell supernatant to evaluate PGE2 levels. PGE2 released from sepsis-related MΜϕs was an approximately 1.5-fold increase in response to GSK101 stimulation 7 days mice versus sepsis at day 1 of therapy (**Figure 3A**). To explore whether activation of PGE2 secretion by MMs affects intestinal contractions under physiological conditions, we applied exogenous PGE2 to the small intestinal segments of mice isolated from each group using the small intestinal organ bath system. We measured changes in spontaneous contraction force and frequency using a dedicated instrument. Compared with the control group, sepsis 1-day small intestine showed a noticeable reduction in spontaneous contractions, as illustrated in **Figures 3B, C**. Likewise, adding exogenous PGE2 (100 μM) to the organ bath system of sepsis 1-day mice significantly increased the spontaneous contraction force. Another study showed that PGE2 differentially affects GI motility by binding to four membrane-bound G protein-coupled E-prostaglandin receptors (EP1–EP4), which can increase or decrease intestinal muscle contraction (29, 30). Our data indicated that the recovery of intestinal motor function significantly increased mRNA levels of Ptger3 in the muscularis externa (**Figure 3D**). Moreover, TRPV4 regulation of gut motility was shown to be dependent on EP3-mediated PGE2 signaling in our immunohistochemistry and western blot results (**Figures 3E–G**). Similarly, we extracted the primary intestinal smooth muscle of sepsis 7 days mice for *in vitro* experiments. Fluo4, a well-established membrane-permeable compound that indicates intracellular Ca2+, and the change of intracellular calcium ion concentration was used to replace the movement of the intestinal tract in animals (31, 32). The laser confocal and incucyte real-time observation results showed that when we used the EP3 antagonist to primary smooth muscle cells in advance, the ability of PGE2 to cause calcium ion release was weakened (**Figures 3H, I**, **Figure S1K**). These results indicated that the contraction of intestinal smooth muscle caused by PGE2 depends on the Ptger3 receptor. Collectively, these studies revealed that macrophage mediated small intestine contraction is PGE2-dependent.

## 4 Discussion

The major findings of our study are as follows: intestinal motor dysfunction owing to KP is associated with damage to the intestinal muscularis neurons; neuronal injury is associated with the infiltration of muscle-infiltrating pro-inflammatory neutrophils; and smooth muscle contraction induced by MΜϕs requires PGE2.

KP is a severe multidrug-resistant pathogen in ICU patients and is associated with high morbidity and mortality owing to limited treatment options (3). KP is characterized by an enhanced inflammatory response with hyper infiltration of neutrophils and macrophages, massive production of pro-inflammatory cytokines, and severe lung injury accompanied by damage to other organs (33). Following the development of pneumonia sepsis, we observed pulmonary and hepatic edema and marked intestinal motility dysfunction. It is generally accepted that GI movement is determined by the synchronized activity of motor neurons in the intestinal SMC, ICC, and ENS. In a cecal ligation and puncture sepsis model, *Li* et al. found that septic small bowel motility dysfunction was associated with marked activation of the IL-17 signaling pathway in the muscularis propria (34). Miao et al. revealed that lipopolysaccharide induced septic mouse model dysmotility was associated with morphological changes in the interstitial cells of ICCs. Meanwhile, magnolol pretreatment significantly accelerated intestinal transit, increased muscle contraction, and prevented ICC morphological changes (35). However, in our study, intestinal motor dysfunction in mice post- KP sepsis was mainly related to the loss of intestinal muscularis neurons. The underlying mechanism involved in neuronal damage is infiltration with pro-inflammatory neutrophils.

Mikkelsen et al. were the first to identify “macrophage-like” cells in the muscularis propria of the small intestine using immunohistochemistry and electron microscopy (36). Muscularis MΦ plays a recognized role in regulating innate immunity; they can also communicate with cells required for motility in the gastrointestinal tract. For example, one study showed that muscularis MΦ increased the production of pro-inflammatory cytokines and was correlated with decreased ICCs in Hirschsprung disease (HSCR) associated with intestinal dysmotility (37). Moreover, EGC-muscularis MΦ crosstalk was observed in intestinal motility dysfunction caused by postoperative ileus, which was associated with muscularis MΦ elevated IL-1β levels (38). However, this study showed that TRPV4 muscularis MΦ promoted GI motility through PGE2, directly interacting with SMC.

However, our study had some limitations. First, the blocking agents we used, including neutrophils or macrophages, are for systemic blocking; knockout mice targeting the intestinal muscularis should be used to be more convincing. Second, our choice of bone marrow-derived neutrophils may be biased due to the low number of neutrophils in the muscle layer to verify cell experiments. We will improve our experimental technology in the future. Third, we did not obtain a perfect rescue result regarding whether exogenous administration of PGE2 after macrophage blockade changes intestinal motility.

In conclusion, this study showed that infiltrating neutrophils, leading to neuronal loss, were significantly associated with small bowel motility dysfunction in mice with KP sepsis. The direct effect of macrophage secreted PGE2 binding to the Ptger3 receptor on intestinal smooth muscle can help the motor function recovery of intestinal neuron damage, which indicates the changes in intestinal dysmotility function after the occurrence of sepsis. In addition, further research should focus on changes in the muscle, nerve cells, and immune cells.

## Data availability statement

The original contributions presented in the study are included in the article/**Supplementary Material**. Further inquiries can be directed to the corresponding author.

## Ethics statement

The animal study was reviewed and approved by the Institutional Animal Care and Use Committee, West China Hospital (study number:20220211010).

## Author contributions

HY, QW and YK conceived and designed the experiments. HY, XF, TL and YL performed the experiments. HY, XF and QX contributed materials/analysis tools. HY, XF and QX analyzed the data. HY wrote the manuscript. HY, YK and WQ reviewed all data and finalized the manuscript. All authors contributed to the article and approved the submitted version.

## Supplementary material

Supplementary Figure 1 Histopathological evaluation between control and Klebsiella pneumonia-septic mice. **(A, C)** Gross and hematoxylin and eosin (HE) staining changes in inflammatory cell infiltration in the whole lung of mice. **(D, F)** Control and sepsis mice clinical scores and score indicator related pictures, and weight loss percent. **(G, H)** HE stains changes in intestine transverse cut muscle thickness in control, sepsis 1, 3, and 7 days and statistical histogram results. **(I, J)** Intestine whole-mount immunofluorescence staining of ICCs and intestinal glial cells and a statistical histogram of mean immunofluorescence intensity and mean immunofluorescence areas. **(K)** Effects of PGE2 and ptger3 antagonists on primary smooth muscle using Incucyte live-view serial photography.
