# Supplementary figures and images for: The macrophages regulate intestinal motility dysfunction through the PGE2 Ptger3 axis during Klebsiella pneumonia sepsis
Source: Front Immunol. 2023 Mar 29;14:1147674. doi: 10.3389/fimmu.2023.1147674 (PMC10090685; doi:10.3389/fimmu.2023.1147674)

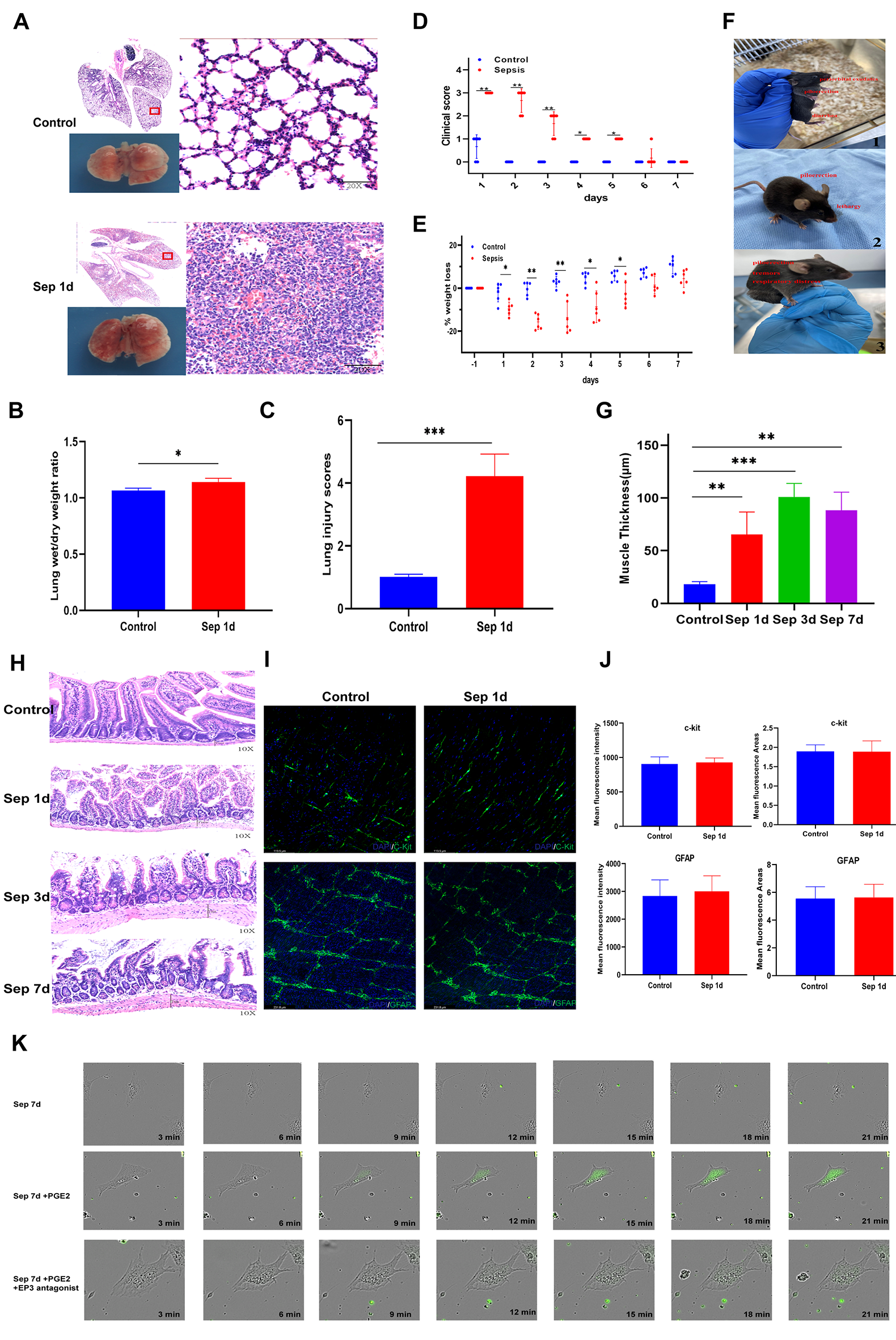

Supplement: Supplementary Figure 1 — Histopathological evaluation between control and Klebsiella pneumonia-septic mice. (A, C) Gross and hematoxylin and eosin (HE) staining changes in inflammatory cell infiltration in the whole lung of mice. (D, F) Control and sepsis mice clinical scores and score indicator related pictures, and weight loss percent. (G, H) HE stains changes in intestine transverse cut muscle thickness in control, sepsis 1, 3, and 7 days and statistical histogram results. (I, J) Intestine whole-mount immunofluorescence staining of ICCs and intestinal glial cells and a statistical histogram of mean immunofluorescence intensity and mean immunofluorescence areas. (K) Effects of PGE2 and ptger3 antagonists on primary smooth muscle using Incucyte live-view serial photography. [file Image_1.tif]
